# Supplementary material for: The Systems Biology Research Tool: evolvable open-source software
Source: BMC Syst Biol. 2008 Jun 29;2:55. doi: 10.1186/1752-0509-2-55 (PMC2446383; doi:10.1186/1752-0509-2-55)
Supplement: Additional file 1 — SBRT Archive. An archive of the current version of the Systems Biology Research Tool. [file 1752-0509-2-55-S1.zip › sbrt-1.4.0/doc/developers_guide/api/sbrt/shell/io/SmoFileSource.html]

SmoFileSource


|  |  |  |
| --- | --- | --- |
| **Overview** \| **Package** \| **Class** \| **Use** \| **Tree** \| **Deprecated** \| **Index** \| **Help** | |  |
| **PREV CLASS**   **NEXT CLASS** | **FRAMES**    **NO FRAMES**     **All Classes** |  |
| SUMMARY: NESTED \| FIELD \| CONSTR \| METHOD | DETAIL: FIELD \| CONSTR \| METHOD |  |


---


## sbrt.shell.io Class SmoFileSource<K,V>

```
java.lang.Object
  sbrt.shell.io.SmoFileSource<K,V>
```

**Type Parameters:**: `K` - the key type.: `V` - the value type.

**All Implemented Interfaces:**: java.util.Iterator<SmoFile<K,V>>

---

``` public class SmoFileSource<K,V> extends java.lang.Object implements java.util.Iterator<SmoFile<K,V>> ```

This class is used to provided a source of serialized map output files.

**Author:**
:   This class was written and documented by
    Jeremiah Wright while in the Wagner lab.

---

**Constructor Summary**

`SmoFileSource(java.util.Iterator<java.lang.String> fileNameSource,
FileFormat fileFormat,
java.util.Set<? extends K> keys)`
  
          Constructs a new source of serialized map output files.


| **Method Summary** | |
| --- | --- |
| `FileFormat` | `getFileFormat()`             Returns the format used to create files of type `SmoFile`. |
| `boolean` | `hasNext()`             Indicates if this iterator can return another file using the `next()` method . |
| `SmoFile<K,V>` | `next()`             Returns the next column-oriented map output file with the next name returned by the file name source. |
| `void` | `remove()`             Throws `UnsupportedOperationException`. |

**Methods inherited from class java.lang.Object**

`clone, equals, finalize, getClass, hashCode, notify, notifyAll, toString, wait, wait, wait`

**Constructor Detail**

### SmoFileSource

```
public SmoFileSource(java.util.Iterator<java.lang.String> fileNameSource,
                     FileFormat fileFormat,
                     java.util.Set<? extends K> keys)
```

:   Constructs a new source of serialized map output files.

    **Parameters:**: `fileNameSource` - the file name source.: `fileFormat` - the file format used to write files.: `keys` - the keys whose values will be written to the files produced by this source. `null` is not allowed. **Throws:**: `java.lang.NullPointerException` - if any argument is `null`.


**Method Detail**

### getFileFormat

```
public FileFormat getFileFormat()
```

:   Returns the format used to create files of type `SmoFile`.

    :   **Returns:**: the format used to create files of type `SmoFile`.

---


### hasNext

```
public boolean hasNext()
```

:   Indicates if this iterator can return another file using the
    `next()` method
    .

    :   **Specified by:**: `hasNext` in interface `java.util.Iterator<SmoFile<K,V>>`
    :   **Returns:**: `true` if this iterator can return another file; `false` otherwise.

---


### next

```
public SmoFile<K,V> next()
```

:   Returns the next column-oriented map output file with the next
    name returned by the file name source.

    :   **Specified by:**: `next` in interface `java.util.Iterator<SmoFile<K,V>>`
    :   **Returns:**: the next column-oriented map output file.

---


### remove

```
public void remove()
```

:   Throws `UnsupportedOperationException`.

    :   **Specified by:**: `remove` in interface `java.util.Iterator<SmoFile<K,V>>`
    :   **Throws:**: `java.lang.UnsupportedOperationException`


---


|  |  |  |
| --- | --- | --- |
| **Overview** \| **Package** \| **Class** \| **Use** \| **Tree** \| **Deprecated** \| **Index** \| **Help** | |  |
| **PREV CLASS**   **NEXT CLASS** | **FRAMES**    **NO FRAMES**     **All Classes** |  |
| SUMMARY: NESTED \| FIELD \| CONSTR \| METHOD | DETAIL: FIELD \| CONSTR \| METHOD |  |


---
